# Apraxia Rehabilitation Using Gesture Training and Sequencing Tasks: A Case Report

**DOI:** 10.7759/cureus.77115

**Published:** 2025-01-07

**Authors:** Tomohiro Eguchi, Jun Takashiba, Shu Morioka

**Affiliations:** 1 Physical Therapy, Chikamori Rehabilitation Hospital, Kochi, JPN; 2 Neurorehabilitation, Kio University, Nara, JPN

**Keywords:** apraxia, gesture training, rehabilitation, sequencing task, stroke

## Abstract

This case report describes the effects of combining gesture training and sequencing tasks to improve apraxia and enhance activities of daily living (ADLs) in a patient with left inferior parietal lobule and temporal lobe damage caused by a cardioembolic stroke. The intervention leveraged the patient's preserved semantic knowledge of tools. A male patient in his late 70s presented with significant impairments in gesture execution and action sequencing while retaining semantic knowledge of tool functions. Beginning on day 13 post-onset, the intervention included gesture training with photographs depicting tool use and sequencing tasks using photo and text cards outlining ADL steps. By discharge on day 147, the patient's Apraxia Screen of TULIA (AST) scores improved from 0 bilaterally to 7 on the right and 9 on the left, and the Functional Independence Measure (FIM) motor score increased from 65 to 87. At follow-up on day 425, AST scores further improved to 10 bilaterally, and the patient achieved independence in basic ADLs. This case demonstrates the efficacy of combining gesture training and sequencing tasks in addressing apraxia and improving functional outcomes, even in the presence of extensive damage to the left inferior parietal lobule. The findings underscore the value of leveraging preserved semantic knowledge in rehabilitation.

## Introduction

Apraxia is a neurological disorder characterized by the inability to execute learned, purposeful movements, despite the absence of motor paralysis, sensory deficits, or significant cognitive impairments. This condition often disrupts activities of daily living (ADLs), particularly due to impairments in tool use and gesture imitation, both of which are critical for functional independence [1-4]. Lesions in the left inferior parietal lobule and the left inferior frontal gyrus are commonly associated with these deficits [5].

Tool use and gesture execution involve visual pathways, notably the ventro-dorsal stream, which is responsible for manipulation and functional knowledge [6,7], and the ventral stream, which supports semantic knowledge of tools [7,8]. Interestingly, studies have shown that patients with dorsal stream damage can successfully grasp familiar tools, suggesting compensatory interactions between these pathways [9].

Emerging therapies such as transcranial direct current stimulation (tDCS) and immersive virtual reality (VR)-based rehabilitation have shown promise in mitigating apraxia, but their application remains limited due to a lack of robust evidence and the need for specialized equipment [10-14]. In contrast, gesture training and strategy-based interventions are accessible and have demonstrated efficacy in improving apraxia and ADL performance in several studies [15-20].

Gesture training is a method aimed at restoring functional movement by using tools and images to facilitate both intransitive and transitive gestures [21]. Strategy training focuses on compensating for apraxia symptoms during ADLs. In this approach, clinicians identify problematic ADLs, select appropriate plans and tools to address them, and guide patients in implementing these plans while correcting errors. During the training process, internal compensation involves verbally articulating the steps of an action, whereas external compensation presents the sequence of actions using pictures or photographs [19,22].

Based on these findings, we hypothesized that (1) leveraging preserved semantic knowledge via the ventral stream could facilitate improvements in gesture execution, even in the presence of dorsal stream damage, and (2) combining gesture training with strategy-based interventions would lead to more effective improvements in ADL performance.

This case report examines a patient with left inferior parietal lobule damage who retained semantic knowledge of tools but exhibited deficits in transitive and intransitive gestures and action sequencing. The intervention combined gesture training and external compensatory strategies to improve action planning and sequencing. We describe the progression of the intervention and evaluate its long-term effects nine months post-intervention.

## Case presentation

The patient was a right-handed Japanese man in his late 70s who had been independent in all ADLs prior to admission. He was capable of driving and worked part-time twice a week. In his leisure time, he enjoyed flower cultivation and farming. His medical history included atrial fibrillation, for which he was receiving anticoagulant therapy, and reduced vision in his right eye due to a prior retinal detachment.

On day 1 of stroke onset, the patient presented with aphasia and right-sided hemiparesis. A computed tomography (CT) scan revealed a hyperdense middle cerebral artery (MCA) sign, consistent with embolism. An electrocardiogram confirmed atrial fibrillation, leading to a diagnosis of cardioembolic stroke. The patient underwent immediate treatment with intravenous tissue plasminogen activator (t-PA) therapy followed by mechanical thrombectomy, resulting in the partial recanalization of the MCA. Although his right-sided hemiparesis showed improvement, severe receptive aphasia persisted.

**Figure 1:**
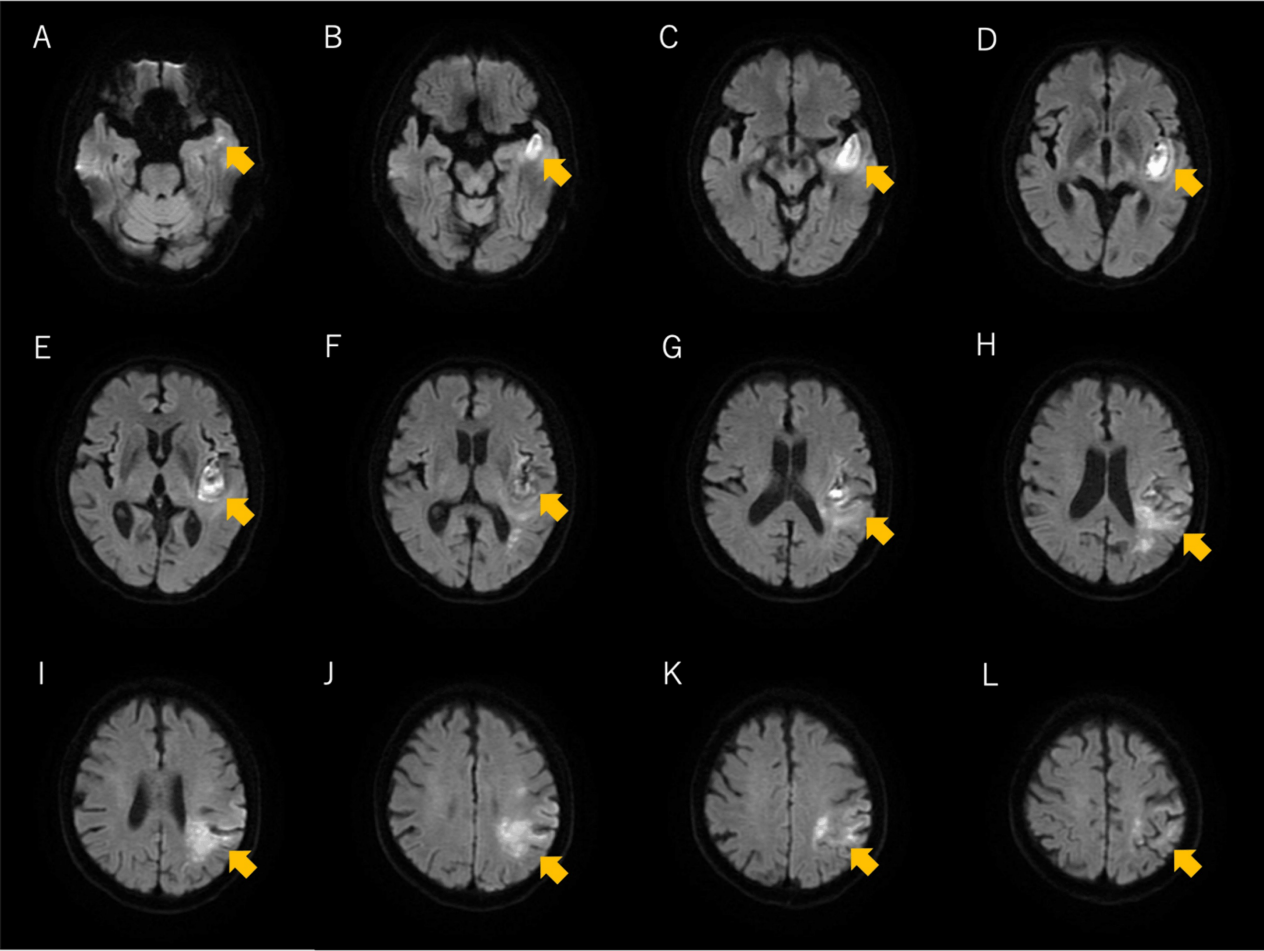
Diffusion-weighted MRI showing acute ischemic stroke lesions Axial DWI on day 12 post-onset reveals hyperintense ischemic lesions (yellow arrows) in the left temporal and parietal lobes. (A-D) Lesions are visible in the posterior superior temporal gyrus (Wernicke's area) and the middle temporal gyrus. (E-H) Involvement extends to the left parietal lobe, specifically affecting the supramarginal and angular gyri, which are linked to praxis and action sequencing. (I-L) Lesions are also observed near the parietal cortex, close to the sensorimotor area, correlating with motor deficits and apraxia symptoms. These findings align with the patient's severe receptive aphasia, apraxia, and motor planning impairments. An MRI was taken on the second day of illness, but the image quality was poor, and the MRI on the 12th day clearly showed an infarct lesion in the parietal lobe and an infarction in the temporal lobe. Therefore, I posted a 12-day MRI scan. MRI: magnetic resonance imaging; DWI: diffusion-weighted imaging

By day 4 post-onset, apraxia was identified as a prominent symptom, manifesting as significant difficulty with object manipulation, including using chopsticks. On day 12, diffusion-weighted magnetic resonance imaging (MRI) revealed infarcts extending from the parietal lobe (supramarginal gyrus and angular gyrus) to the temporal lobe (Wernicke's area, superior temporal gyrus, and parts of the temporal pole) (Figure 1). On day 13, the patient was transferred to our specialized rehabilitation hospital for post-stroke recovery.

Rehabilitation interventions were initiated at the referring hospital, where occupational therapy (OT) and speech therapy (ST) began on day 4 post-onset, followed by physical therapy (PT) on day 5. According to the referral note, the patient demonstrated significant apraxia when manipulating objects such as chopsticks and combs. Although specifics of the acute-phase interventions were not fully documented, rehabilitation was promptly resumed after his transfer to our hospital on day 13. A team comprising a physiotherapist, an occupational therapist, and a speech therapist worked collaboratively to address his dysfunction, and targeted PT intervention focusing on apraxia was initiated on day 22 post-onset.

Written informed consent was obtained from the patient and his family for the publication of this case report and accompanying images. To protect privacy, all personally identifiable information has been minimized.

Admission assessment (days 13-22 post-onset)

Upon admission, the patient exhibited severe Wernicke's aphasia. Performance on the Standard Language Test of Aphasia (SLTA) showed 0% accuracy in auditory language comprehension. In reading comprehension, accuracy was 70% for kanji words and 80% for kana words, but 0% for short sentences. Apraxia further impaired the patient's ability to follow verbal instructions, limiting the scope of detailed assessments. The patient consistently followed the therapists' instructions, participated fully in assigned tasks without refusal, and initiated questions to clarify the therapeutic exercises. These behaviors indicated high engagement and a willingness to cooperate throughout the rehabilitation sessions.

Physical Function

The patient's Brunnstrom stages for the right upper limb, fingers, and lower limb were all at level V or higher, indicating no significant motor paralysis. Sensory function was intact, as confirmed during motion analysis. The Berg Balance Scale (BBS) score was 48 out of 56. Generally, a score of 41-56 indicates a low fall risk, 21-40 indicates a moderate fall risk, and 0-20 indicates a high fall risk. Therefore, a score of 48 suggests that the patient had near-normal balance but still required some caution. The Functional Independence Measure (FIM) total score was 75, comprising 65 points for motor tasks and 10 points for cognitive tasks.

Apraxia Assessment

The Apraxia Screen of TULIA (AST) is a shortened version of the TULIA test, designed for the rapid assessment of apraxia. It comprises 12 items: one item for imitation of meaningless intransitive gestures, one for imitation of intransitive gestures, five for imitation of transitive gestures, two for pantomime of intransitive gestures, and three for pantomime of transitive gestures. Each side is scored out of 12 points, with scores below 9 indicating apraxia and below 5 indicating severe apraxia. In this case, the patient scored 0 out of 12 on both sides, which confirms a severe level of apraxia. This deficit significantly affected the patient's ability to perform gestures and action sequencing, particularly in the context of ADLs.

ADL Performance

Observations of the patient's ADL performance revealed various challenges. During meals, he struggled to use a spoon with his right hand, often relying on his left hand under supervision. In oral care, he mistakenly dispensed toothpaste into a cup rather than onto a toothbrush; however, once the toothpaste was correctly applied, he could complete the task independently. Dressing was achievable with supervision, though he occasionally required additional time to manage zippers and buttons. Regarding toileting, the patient was able to manage clothing but needed assistance for thorough wiping after defecation and for changing diapers when soiled. During bathing, errors in task sequencing were noted, such as confusion in the order of body washing and shampooing, as well as difficulties in operating soap bottles.

Intervention

**Figure 2:**
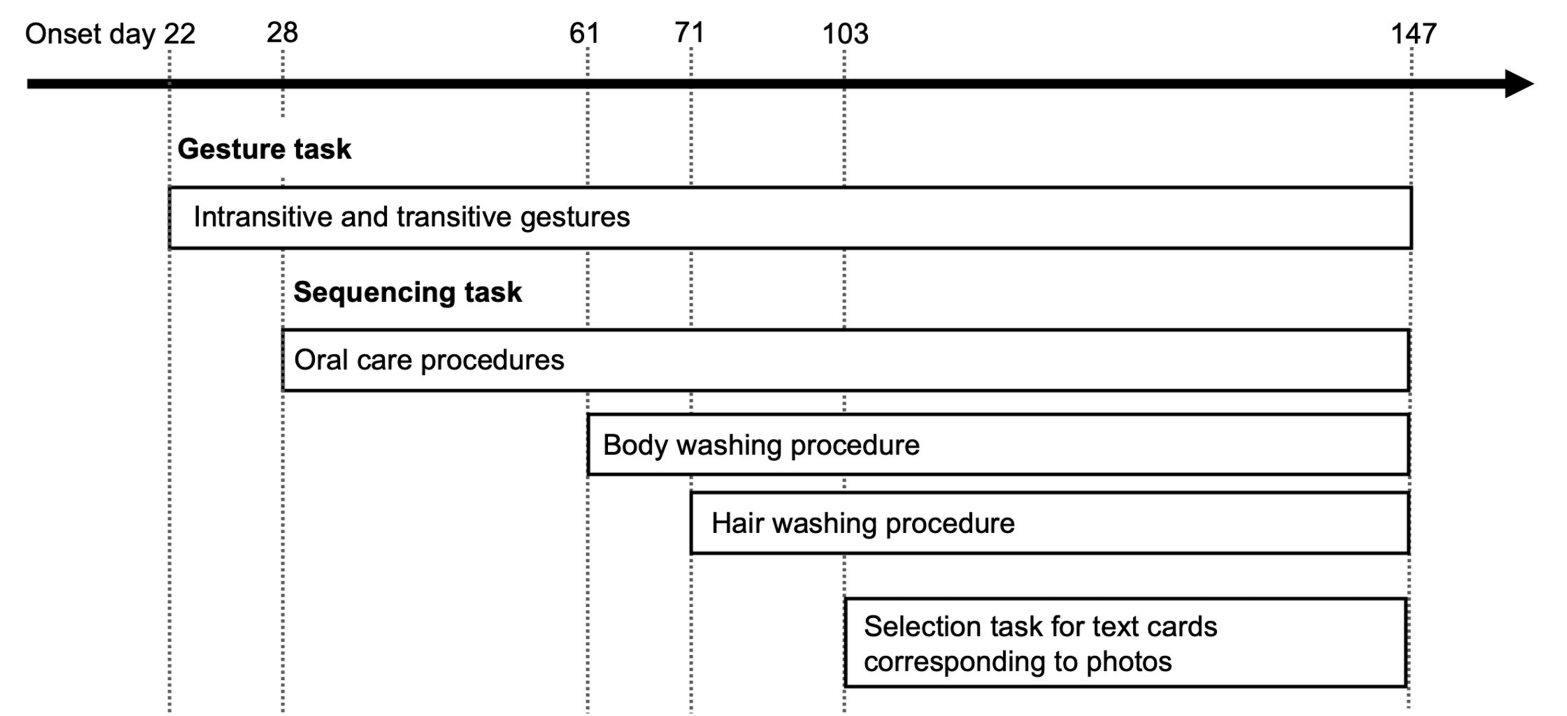
Timeline of gesture training and sequencing tasks during rehabilitation The timeline illustrates the structured rehabilitation program from day 22 to day 147 post-onset. Gesture training, starting on day 22, targeted intransitive and transitive gestures essential for ADLs. Sequencing tasks were introduced on day 28, focusing first on oral care, followed by body washing (day 61) and hair washing (day 71). On day 103, a selection task combining text cards and photographs was added to further improve cognitive integration and task execution. This timeline highlights the progression of interventions aimed at enhancing gesture performance, action sequencing, and functional independence. ADLs: activities of daily living

The patient underwent a comprehensive intervention program combining gesture training and strategy-based sequencing tasks for 20-30 minutes per day. This intervention aimed to alleviate apraxia symptoms by utilizing the patient's preserved semantic knowledge of tools and addressing deficits in action planning and sequencing, particularly in ADLs. The intervention content and timeline are summarized in Figure 2.

Gesture Training

**Figure 3:**
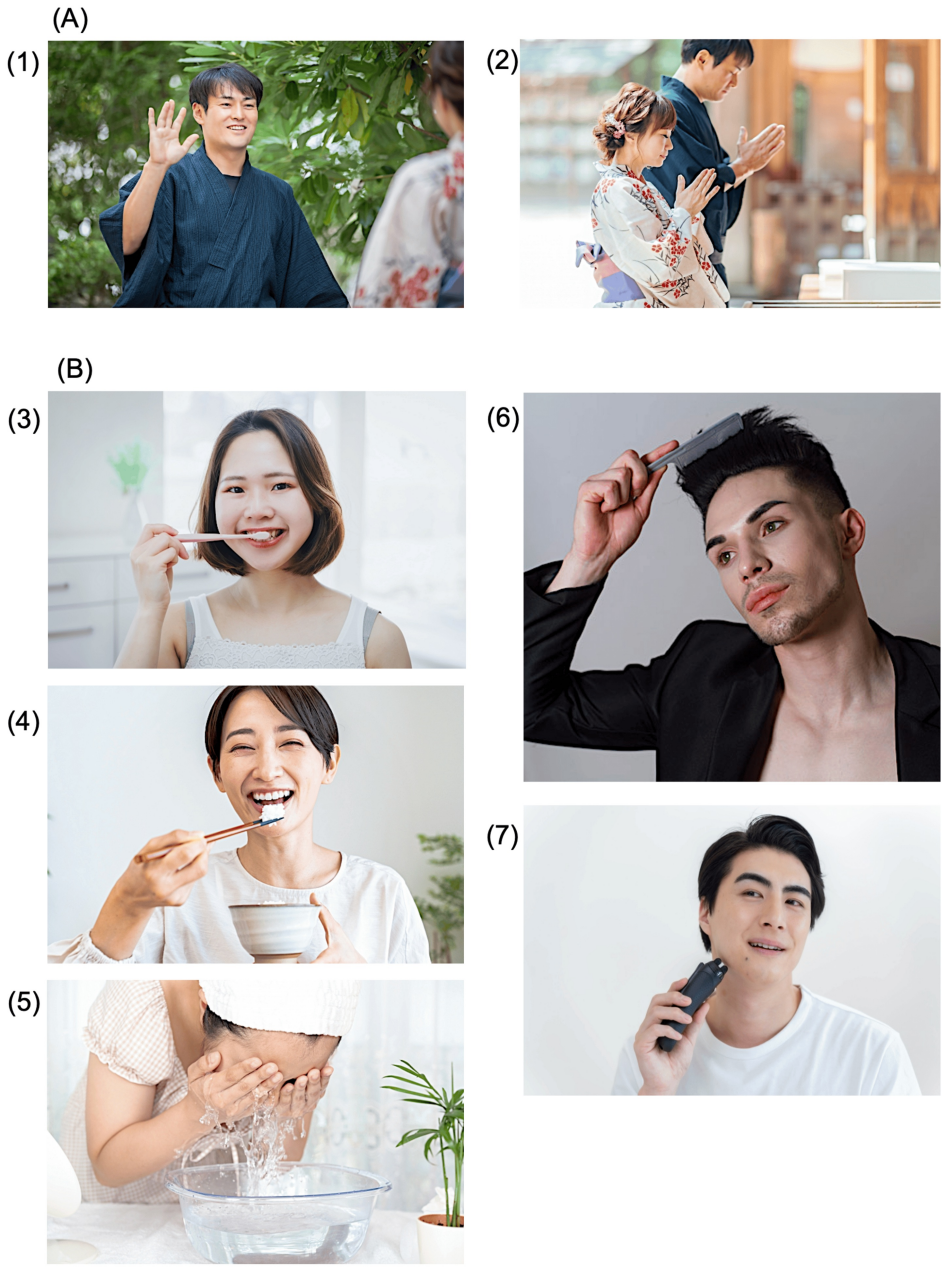
Photographs of intransitive and transitive gestures used in gesture training (A) Intransitive gestures involve tool-free movements, such as waving (1) and bowing (2). (B) Transitive gestures require tools and are critical for ADLs, including brushing teeth (3), eating with chopsticks (4), washing the face (5), combing hair (6), and shaving with an electric razor (7). Patients performed these gestures by referencing the photographs. Errors were corrected through therapist demonstrations, imitation, or stepwise manual guidance to ensure accuracy. Emphasis was placed on transitive gestures to reinforce ADL-related motor planning and facilitate functional recovery. Images are used under license, and no additional attribution is required as per photoAC's terms of use. Photo source:photoAC (https://www.photo-ac.com/) ADLs: activities of daily living

Gesture training commenced on day 22 post-onset. In total, 13 photographs were prepared for training, comprising three intransitive gestures (e.g., waving hands, bowing) and 10 transitive gestures (e.g., brushing teeth, eating with chopsticks, washing the face, combing hair, shaving with an electric razor). However, Figure 3 presents seven representative examples from this set: photos 1-2 (intransitive gestures) and photos 3-7 (selected transitive gestures). Patients were instructed to imitate each gesture based on the photographs, and any errors were corrected through demonstrations, imitation, or stepwise manual guidance to ensure accurate execution.

During each session, the patient was guided to execute gestures based on the photographs. In cases where errors occurred, the therapist provided corrective feedback, starting with imitation of the therapist's movements. If imitation remained challenging, stepwise manual guidance was applied to facilitate proper execution.

Sequencing Tasks

The sequencing tasks were designed to identify real-life sequencing difficulties and introduce targeted exercises to address them. Sequencing tasks were introduced on day 28 post-onset, beginning with oral care. The patient was tasked with arranging three photograph cards in the correct order: "apply toothpaste to the toothbrush," "brush teeth," and "rinse mouth." Starting on day 51 post-onset, text cards representing the same sequence were added to further enhance cognitive integration of the steps.

On day 61 post-onset, sequencing tasks for body washing were introduced. The patient used text cards to sequence the following steps: "apply soap to a towel," "wash the body," and "rinse off the foam with a shower." From day 71 post-onset, hair washing tasks were added, with steps including "apply shampoo to the hand," "wash the hair," and "rinse off the foam with a shower."

**Figure 4:**
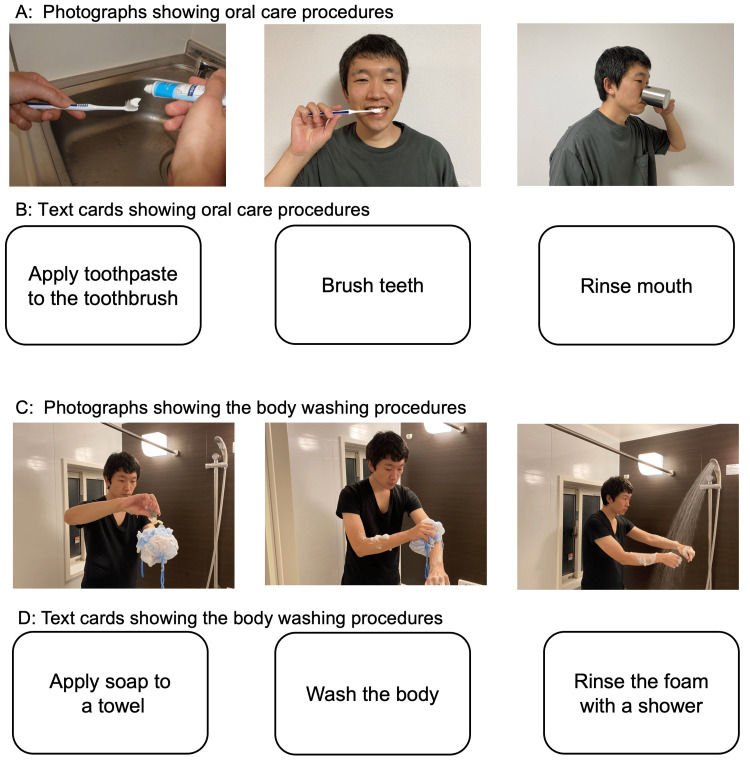
Photographs and text cards used in sequencing and text card matching tasks Photographs and text cards were utilized in sequencing and text card matching tasks to improve action planning and execution. (A, B) Oral care tasks, including steps such as "apply toothpaste to the toothbrush," "brush teeth," and "rinse mouth." (C, D) Body washing tasks, illustrating steps like "apply soap to a towel," "wash the body," and "rinse off the foam with a shower." In the sequencing task, patients arranged the photographs and text cards in the correct order to match procedural steps. In the text card matching task, patients matched individual text cards to corresponding photographs presented on a tabletop. These tasks reinforced stepwise cognitive integration and functional sequencing of ADLs. All photographs and text cards were created by the authors. ADLs: activities of daily living

In this case, the patient's difficulty with the text card instructions for washing hair and body necessitated expanding the intervention from day 103 post-onset to include photographs illustrating each step of these tasks. Gesture training was further integrated into this phase, requiring the patient to identify and execute gestures corresponding to the photographs and to match text cards to the appropriate steps (Figure 4).

Timeline and Duration

All interventions were conducted daily and continued until the patient's discharge on day 147 post-onset. The combination of gesture training and sequencing tasks aimed to improve gesture execution, enhance action sequencing, and support ADL performance, thereby promoting greater functional independence.

Progress

Changes in the AST and FIM scores are summarized in Table 1. The intervention was divided into three stages, early, mid, and late, based on key milestones in gesture execution, sequencing tasks, and ADL performance.

**Table 1:** AST and FIM scores from initial assessment to post-discharge follow-up The table summarizes the patient's AST and FIM motor scores from day 22 post-onset to follow-up on day 425. Pantomime testing from day 53 included written instructions alongside verbal instructions to accommodate the patient's superior written comprehension. AST scores demonstrated marked improvement, particularly with transitive gesture tasks and pantomime execution, while FIM motor scores reflected steady recovery of ADL independence, culminating in a total score of 111 at discharge. AST: Apraxia Screen of TULIA; FIM: Functional Independence Measure; ADL: activity of daily living

Assessments		Onset day 22	Onset day 53	Onset day 81	Onset day 109	Onset day 147	Onset day 425
AST							
Imitation meaningless (1)	Rt/Lt	0/0	0/0	0/0	0/0	0/1	1/1
Imitation intransitive (1)	Rt/Lt	0/0	0/0	0/0	1/1	1/1	1/1
Imitation transitive (5)	Rt/Lt	0/0	2/2	2/3	4/5	4/4	4/4
Pantomime intransitive (2)	Rt/Lt	0/0	0/0	1/1	1/1	1/1	1/1
Pantomime transitive (3)	Rt/Lt	0/0	1/1	2/1	1/1	1/2	3/3
Total score (12)	Rt/Lt	0/0	3/3	5/5	7/8	7/9	10/10
FIM							
Motor FIM (91)		65	78	83	84	87	-
Cognitive FIM (35)		10	20	22	22	24	-
Total score (126)		75	98	105	106	111	-

Early Stage (Days 22-53 Post-Onset)

At the beginning of gesture training, the patient made errors in almost all gestures. He struggled to execute appropriate gestures even when presented with photographs, and imitating the therapist's movements did not consistently resolve these errors. Manual guidance was frequently required to facilitate proper gesture execution, although the patient could temporarily reproduce correct gestures immediately after manual correction.

By day 53 post-onset, notable improvements were observed. The patient was able to correctly perform six of the 13 gestures without assistance. The number of gestures requiring imitation decreased to four, and those requiring manual correction were reduced to three. AST scores improved to 3 bilaterally, particularly in the imitation and pantomime of transitive gestures. Sequencing tasks for oral care initially relied on guessing; however, after receiving corrective feedback, the patient was able to arrange the photograph cards correctly without hesitation.

In the SLTA, auditory language comprehension improved to 50% for single words, while other categories remained at 0%. Reading comprehension reached 100% for both kanji and kana words and 80% for short sentences. Sequencing tasks incorporating text cards for oral care, introduced during this period, also yielded correct responses. The FIM score improved to 78 for motor tasks and 20 for cognitive tasks. In ADL performance, the patient became independent in eating with a spoon using his right hand, corrected errors in oral care sequencing, and achieved independence in dressing.

Mid Stage (Days 54-81 Post-Onset)

By day 81 post-onset, the number of gestures performed correctly (six), those requiring imitation (four), and those requiring manual correction (three) remained the same, although the time needed to achieve a correct response had decreased. Sequencing tasks involving photograph cards for oral care were consistently performed correctly, though minor hesitation persisted when using text cards. Newly introduced sequencing tasks for body washing and hair washing using text cards revealed continued errors.

AST scores improved to 5 bilaterally, and FIM scores increased to 83 for motor tasks and 22 for cognitive tasks. The patient became capable of eating with chopsticks and independently managing urinary pad replacement. However, errors during bathing tasks persisted, including selecting the wrong items (e.g., mistaking shampoo for body soap), inverting pump bottles, and using inappropriate methods (e.g., washing the body with shampoo). To mitigate these issues, the intervention was modified by introducing a single cleansing agent suitable for both body and hair washing and replacing pump bottles with one-touch cap bottles to simplify handling. These adjustments significantly reduced item selection and bottle handling errors.

Late Stage (Days 82-109 Post-Onset)

By day 109 post-onset, the patient was able to correctly perform 10 gestures. The patient required imitation for only three gestures, with manual corrections rarely necessary. Spatial errors, such as palm orientation, decreased significantly, and the patient began self-correcting these errors. Sequencing tasks involving photograph cards for all action sequences were performed correctly; however, errors persisted in text card sequencing.

AST scores had improved to 7 on the right and 8 on the left, while FIM scores reached 84 for motor tasks and 22 for cognitive tasks. Errors in bottle handling during bathing were eliminated after introducing simplified bottle designs. However, sequencing errors in body washing tasks persisted, indicating ongoing deficits in action planning and execution despite overall functional gains. Because daily tracking of errors was not feasible, we relied on periodic evaluation recordings to gauge gesture errors and whether they were correctable by imitation or required hands-on assistance. Although we did not precisely measure the time per session, the total completion time for the 13 gesture training items dropped from over 10 minutes initially to under five minutes by the end of the program, suggesting both fewer errors and greater efficiency. Nonetheless, the persistence of sequencing errors underscores the need for continued strategies targeting higher-order motor planning.

Evaluation at discharge (day 147 post-onset)

At discharge, the patient demonstrated significant improvements in both gesture execution and sequencing tasks. In the gesture training tasks, the patient correctly performed 10 gestures; only three required imitation, and none necessitated manual correction. Sequencing tasks using photographs were completed without hesitation, while text card sequencing tasks were performed accurately after careful consideration. AST scores improved to 7 on the right and 9 on the left, and the FIM score increased to 87 for motor tasks and 24 for cognitive tasks. The patient achieved independence in all ADLs except for bathing, which was performed with minimal errors.

Language assessments also showed notable progress. In the SLTA, auditory language comprehension accuracy improved to 80% for single words and 70% for short sentences. Reading comprehension accuracy for short sentences reached 60%.

Physical function evaluations revealed no signs of motor paralysis or sensory impairments. The BBS score was 54, indicating that the patient could walk independently without assistive devices.

From day 17 post-onset, the patient exhibited significant emotional distress, frequently expressing frustration related to his aphasia, difficulty manipulating objects, and feelings of shame over toileting issues. By day 22, these symptoms had intensified, with the patient often looking down when spending time alone. As these signs of severe stress persisted, the attending physician prescribed a selective serotonin reuptake inhibitor (SSRI) starting on day 25, which continued until day 119. Despite his emotional challenges, the patient consistently followed therapists' instructions and participated fully in assigned tasks without refusal, demonstrating high engagement and a strong willingness to cooperate throughout the rehabilitation sessions.

Post-discharge follow-up (day 425 post-onset)

Following discharge, the patient continued weekly outpatient rehabilitation, consisting of OT (until day 215 post-onset) and ST (until day 425 post-onset). The post-discharge interventions did not include gesture training or sequencing tasks but focused on attention and construction tasks, writing exercises in OT, and communication-focused interventions in ST, such as comprehension, expression, and conversational training.

By day 425 post-onset, AST scores further improved to 10 bilaterally. According to interviews with family members, the patient achieved full independence in bathing and all other basic ADLs. He resumed farm work as a leisure activity under the supervision of family members, successfully using basic tools such as a hoe but facing challenges with operating complex machinery.

## Discussion

This case highlights the effectiveness of tailored gesture training and sequencing tasks in improving apraxia symptoms and ADL independence in a patient with infarction in the inferior parietal lobule. The observed deficits in gesture execution and action sequencing align with findings that damage to the inferior parietal lobule disrupts action specification, execution, and generation, ultimately leading to tool-use impairments and challenges in daily activities [23].

Interpretation of observed improvements

The intervention resulted in significant improvements in AST scores (from 0 to 7 on the right and 0 to 9 on the left) and FIM motor scores (from 65 to 87). Errors during gesture training and sequencing tasks decreased after the targeted PT intervention focused on apraxia, corresponding with the rise in AST and FIM scores. Although this case study does not establish a definitive causal relationship, the findings suggest that reducing apraxia symptoms may contribute to broader functional gains. Furthermore, the improvement in AST scores persisted over a nine-month follow-up period, indicating that the intervention strategies likely played an important and lasting role.

The multidisciplinary rehabilitation program, incorporating PT for apraxia, OT for ADL training, and ST for communication, likely contributed to these outcomes. However, the compensatory strategies tailored to the patient's specific deficits, particularly the use of visual and textual tools, proved essential in addressing gesture and sequencing impairments.

Comparison with previous studies

The results align with Smania et al. [17], who demonstrated significant ADL improvements following 25 hours of gesture training over 30 sessions. In this case, similar outcomes were achieved with approximately 20 hours of intervention over 120 days. The earlier initiation of rehabilitation (day 22 post-onset) compared to Smania et al.'s average of 10 months post-onset underscores the benefits of early intervention, though the role of spontaneous recovery must be considered.

Unlike the three-tiered approach described by Smania et al., which included actual tool use, tool-use photographs, and photographs of tools alone, this case primarily utilized tool-use photographs. The patient's ability to manipulate tools early in recovery allowed the intervention to focus on higher-level tasks, bypassing the need for lower-tiered strategies.

The strategy training framework outlined by van Heugten et al. [22], emphasizing compensatory strategies for action planning, object selection, and error correction, was adapted to suit the patient's specific needs. External compensatory strategies, such as photographs and text cards, were prioritized over internal strategies (e.g., verbalization) due to the patient's severe aphasia. Despite these modifications, the intervention duration was comparable to van Heugten et al.'s approach (approximately 20 hours) and resulted in similar improvements in action planning and object selection.

Neurological mechanisms underlying improvements

The ventral cortical visual pathway, which processes semantic knowledge, played a compensatory role for dorsal pathway deficits in this case, particularly in tool-use tasks [9]. Despite partial damage, the temporal pole, which is essential for integrating object meanings from visual and linguistic information, supported the patient's preserved semantic knowledge. This compensation facilitated improved transitive gesture performance, as reflected in the patient's AST scores [24].

Damage to the supramarginal gyrus, a region within the inferior parietal lobule, often results in persistent deficits in meaningless gesture imitation, pantomime generation, and pantomime conceptualization due to its limited compensatory capacity [25]. However, the tailored combination of gesture training and sequencing tasks, in this case, enabled significant recovery in gesture performance and ADL independence, even with extensive parietal lobe damage.

Sequencing tasks, particularly those incorporating text cards, leveraged motor learning mechanisms. Reading action-related verbs, such as "brush teeth," activates the primary motor cortex, thereby reinforcing motor learning through mental simulation [26,27]. This mechanism likely contributed to a reduction in sequencing errors and improved action comprehension.

The integration of perceptual and motor components was another critical factor in the patient's recovery. Observing tool-use photographs and generating corresponding gestures created a synergistic effect, enhancing perceptual-motor integration, as described by Pazzaglia and Galli [28]. Building on the work of Smania et al. [21], this synergy supported the recovery process. Furthermore, intentional observation of movements has been shown to increase motor area excitability, further enhancing motor control and gesture performance, as noted by Sale et al. [29].

The mirror neuron system, particularly in the intact inferior frontal gyrus, also played a crucial role in the patient's recovery. This system is vital for understanding and imitating observed actions, integrating visual inputs with motor responses [30]. The intact functionality of this region likely contributed to the improvements in gesture execution and ADL performance observed in this case.

Practical implications

This case underscores the importance of leveraging preserved neurological functions and tailoring rehabilitation strategies to patient-specific deficits. Early intervention, gesture training focused on transitive gestures, and the use of visual aids and text cards proved effective in addressing apraxia symptoms. These findings suggest that personalized approaches can maximize recovery potential even in cases of significant neurological damage.

Limitations

This case report has several limitations that must be acknowledged. First, the influence of spontaneous recovery cannot be entirely excluded, as the intervention was initiated early in the post-stroke period (day 22 post-onset). While the sustained improvements observed over the follow-up period suggest the effectiveness of the intervention, it is challenging to differentiate its contributions from natural recovery processes.

Second, the patient underwent a comprehensive rehabilitation program that included PT, OT, and ST. This multidisciplinary approach, while essential for the overall recovery, complicates the isolation of the specific effects of gesture training and sequencing tasks.

Lastly, this is a single-case report, and its findings may not be generalizable to all patients with apraxia or similar lesions. Future research involving larger sample sizes and controlled studies is necessary to validate the efficacy of these intervention strategies and to explore their broader applicability.

## Conclusions

The rehabilitation of apraxia requires a thorough evaluation of preserved neurological functions and the development of tailored intervention strategies based on these assessments. In this case, a combination of gesture tasks leveraging the patient's semantic knowledge of tools and sequencing tasks aimed at facilitating action planning resulted in significant improvements in apraxia symptoms and ADL performance. Notably, these effects were sustained over the long term.

This case underscores the importance of personalized intervention strategies that address the specific needs and preserved abilities of individuals with apraxia. Tailored combinations of interventions, such as those implemented in this case, are critical for optimizing recovery outcomes and promoting functional independence in daily living.
